# Pharmacophore Modelling and 3D-QSAR Studies on N^3^-Phenylpyrazinones as Corticotropin-Releasing Factor 1 Receptor Antagonists

**DOI:** 10.1155/2012/452325

**Published:** 2012-05-31

**Authors:** Paramjit Kaur, Vikas Sharma, Vipin Kumar

**Affiliations:** Institute of Pharmaceutical Sciences, Kurukshetra University, Haryana, Kurukshetra 136119, India

## Abstract

Pharmacophore modelling-based virtual screening of compound is a ligand-based approach and is useful when the 3D structure of target is not available but a few known active compounds are known. Pharmacophore mapping studies were undertaken for a set of 50 N^3^-phenylpyrazinones possessing Corticotropin-releasing Factor 1 (CRF 1) antagonistic activity. Six point pharmacophores with two hydrogen bond acceptors, one hydrogen bond donor, two hydrophobic regions, and one aromatic ring as pharmacophoric features were developed. Amongst them the pharmacophore hypothesis AADHHR.47 yielded a statistically significant 3D-QSAR model with 0.803 as *R* ^2^ value and was considered to be the best pharmacophore hypothesis. The developed pharmacophore model was externally validated by predicting the activity of test set molecules. The squared predictive correlation coefficient of 0.91 was observed between experimental and predicted activity values of test set molecules. The geometry and features of pharmacophore were expected to be useful for the design of selective CRF 1 receptor antagonists.

## 1. Introduction

Anxiety and depression are among the most common disorders seen in medical practice. The coexistence of anxiety and depression with medical illness is a topic of considerable clinical and research interest [1]. Depression is a serious mental health problem, with significant consequences in terms of human suffering, lost productivity, and even loss of life [2]. Corticotropin-releasing factor 1 (CRF 1) receptor antagonists have been sought since the stress-secreted peptide (adrenocorticotropin-releasing hypothalamic peptide) was isolated in 1981. Although evidence is mixed concerning the efficacy of CRF 1 receptor antagonist as antidepressants, CRF 1 receptor antagonist might be novel pharmacotherapies for anxiety and addiction [3]. Two well-characterized receptor subtypes, CRF 1 and CRF 2, have been identified. These G-protein-coupled receptors are widely distributed throughout the central and peripheral nervous systems [4]. Clinical evidence supports the hypothesis that overproduction of CRF 1  may underlie the pathology of depression, anxiety, and stress-related disorders and suggests that CRF 1 receptor antagonists could be useful for the treatment of these conditions [5].

To reduce the overall cost associated with the discovery and development of a new drug, the computer-aided molecular design methods have been identified as the most promising candidates to focus on the experimental efforts in modern medicinal chemistry. Pharmacophore mapping is one of the major elements of drug design in the absence of structural data of the target receptor. With the aim of providing useful pharmacophoric information for the future efforts in the development of more potent molecules in the series of phenylpyrazinones and to get insight into the structural and molecular properties, the ligand-based 3D-QSAR study was performed using pharmacophore techniques with PHASE module from Schrodinger, New York [6, 7].

## 2. Experimental

### 2.1. Dataset

**Figure 1:**
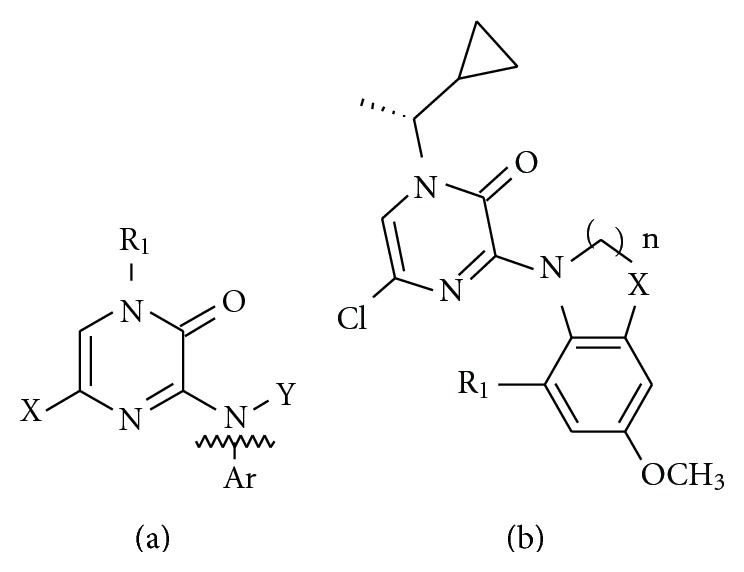
Basic structures of N^3^-phenylpyrazinones.

The *in vitro* biological data of a series of 50 phenylpyrazinones as CRF 1 receptor antagonists were used for the present studies [6]. The CRF 1 antagonistic activity was expressed as IC_50_, that is, concentration in *μ*m required for 50% inhibition of enzyme activity. The dataset was divided randomly into training set and test set by considering the 70% of the total molecules in the training set and 30% in the test set. The basic structures of the N^3^-phenylpyrazinones are shown in Figure 1, and various substituents are listed in Table 1. Thirty-five molecules forming the training set were used to generate pharmacophore model and prediction of the activity of test set (15 compounds) molecules was used as a method to validate the proposed models.

### 2.2. PHASE Methodology

The 3D-QSAR studies were carried out using PHASE [8–10] version 3.0 implemented in the Maestro 8.5 molecular modeling package from Schrodinger, Molecular Modeling Interface Inc., LLC, New York, NY USA. Phase is a versatile product for pharmacophore perception, structure alignment, activity prediction, and 3D database searching. Phase provides support for lead discovery, SAR development, lead optimization, and lead expansion. Phase may also be used as a source of molecular alignments for third-party 3D-QSAR programs. Phase is well suited to drug discovery projects for which no receptor structure is available. Phase utilizes fine-grained conformational sampling and a range of scoring techniques to identify common pharmacophore hypotheses, which convey characteristics of 3D chemical structures that are purported to be critical for binding. A given hypothesis may be combined with known activity data to create 3D-QSAR models that identify overall aspects of molecular structure that govern activity. These models may be used in conjunction with the hypothesis to mine a 3D database for molecules that are most likely to exhibit strong activity toward the target [11].

### 2.3. Preparing Ligands

LigPrep [12] was used to attach hydrogen, converts 2D structures to 3D, generates stereoisomer, and, optionally, neutralizes charged structures or determines the most probable ionization state at user-defined pH. All the structures were ionized at neutral pH 7. Conformers for each ligand were generated using ConfGen by applying OPLS-2005 force field method [13, 14] with implicit GB/SA distance-dependent dielectric solvent model at cutoff root mean square deviation (RMSD) of 1 (MacroModel 9.6 2010) with 1,000 iterations using water as solvent.

### 2.4. Pharmacophore Hypothesis Generation

PHASE can identify the spatial arrangements of functional groups that are common and essential for the biological activity of the ligands under investigation [10, 15]. The most dominating features, hydrogen bond acceptor (A), hydrogen bond donor (D), hydrophobic group (H), and negatively charged group (N) were defined by a set of chemical structural patterns with the requirement that all five match. Pharmacophore-matching tolerance was set to 1 A°. Hypotheses were generated by a systematic variation of number of sites and the number of matching active compounds. Common pharmacophore hypotheses (CPH) were considered, which indicated at least five sites common to all molecules. Further, the best CPH was selected depending on the survival score, until at least one hypothesis was found and scored successfully. The hypotheses were scored using default parameters for site, vector, volume, selectivity, number of matches, and energy terms. The regression analysis was performed by constructing a series of models with an increasing number of PLS factors. Pharmacophore-based 3D-QSAR models were generated for the hypotheses using the 35 member training set with three PLS factors and a grid spacing of 1 A°. The evaluation of generated CPHs was performed by correlating the observed and the estimated activity for the training set of 35 molecules. PLS analyses were performed in which a series of models were constructed with an increasing number of PLS factors. Score hypotheses step was employed to align the actives to the hypotheses and calculate the score for the actives. CPHs of significant statistical values were selected for molecular alignments.

### 2.5. Validation of Pharmacophore Model

For accurate and reliable predictions of biological activities of new compounds, the main target was to develop QSAR models, which were statistically robust both internally as well as externally. The data set was divided into a training set and a test set as external validation is considered to be a conclusive proof for judging predictability of a model. The training set was used to generate pharmacophore model and prediction of the activity of test set was used as a method to validate the proposed models. The robustness of the developed pharmacophore hypotheses was internally validated by statistical parameters, that is, squared correlation coefficient (*R* ^2^) and variance ratio (*F*). Validation is a crucial aspect of pharmacophore design, particularly when the model is built for the purpose of predicting activities of molecules in external test series [16]. In the present case, the developed pharmacophore model was externally validated by predicting the activity of test set molecules. The correlation between the experimental and predicted activities of the test set molecules was determined.

## 3. Results and Discussion

CRF 1 receptor antagonists may offer therapeutic potential for the treatment of diseases resulting from elevated levels of CRF 1 such as anxiety and depression. Efforts to identify structurally diverse CRF 1 receptor antagonists led to the discovery of pyrazinone-based compounds and it was found that CRF 1 receptor binding affinity was improved when the branching point was on the carbon atom bonded directly to the pyrazinone ring [6]. Ligand-based drug design relies on knowledge of other molecules that bind to the biological target of interest. These molecules may be used to derive a pharmacophore which defines the minimum necessary structural characteristics a molecule must possess in order to bind to the target [7]. In other words, a model of the biological target is built based on the knowledge of what binds to it and this model in turn may be used to design new molecular entities that interact with the target. 

Thirty-five molecules forming the training set were used to develop the pharmacophore models. The pharmacophoric features selected for creating sites were hydrogen bond acceptor (A), hydrogen bond donor (D), hydrophobic region (H), and aromatic ring (R). Pharmacophore models containing four to six features were generated. The four and five featured pharmacophore hypotheses were rejected due to low value of survival score (less than 2.5), as they were unable to define the complete binding space of the selected molecules. Six featured pharmacophore hypotheses were selected and subjected to stringent scoring function analysis. 

The results of six featured pharmacophore hypotheses, labeled AADHHR.47, ADHHHR.203, DHHHRR.611, and AAHHHR.19, are presented in Table 2. The first hypothesis AADHHR.47 is the best hypothesis in this study, characterized by highest survival score (2.980) and best regression coefficient (0.803). 

**Figure 2:**
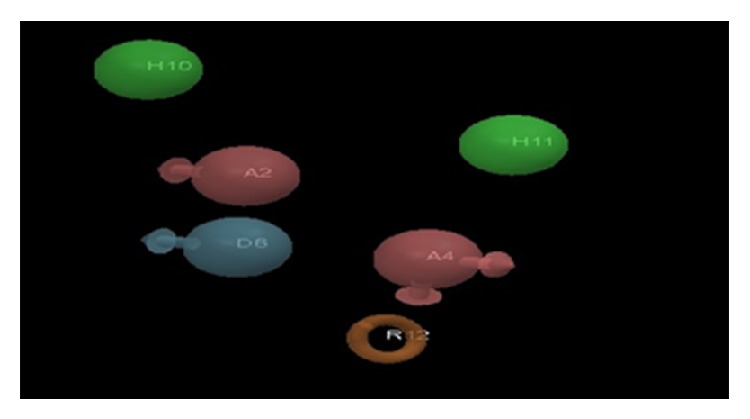
PHASE-generated pharmacophore model AADHHR.47 illustrating hydrogen bond acceptor (A2, A4; pink), hydrogen bond donor (D6; blue), hydrophobic region (H10, H11; green), and aromatic ring (R12; orange) features with distances (in Å) between different sites of AADHHR.47.

The pharmacophore hypothesis AADHHR.47 is presented in Figure 2. The features represented by this hypothesis are two hydrogen bond acceptors (A), one hydrogen bond donor (D), two hydrophobic regions (H), and one aromatic ring (R). The distances and angles between different sites of AADHHR.47 are given in Tables 3 and 4, respectively. 

For each ligand, one aligned conformer based on the lowest RMSE of feature atom coordinates from those of the corresponding reference feature was superimposed on AADHHR.47. The fitness scores for all ligands were observed on the best scored pharmacophore model AADHHR.47. The greater the fitness score, the greater the activity prediction of the compound. The fit function does not only check if the feature is mapped or not but also contains a distance term, which measures the distance that separates the feature on the molecule from the centroid of the hypothesis feature. 

**Figure 3:**
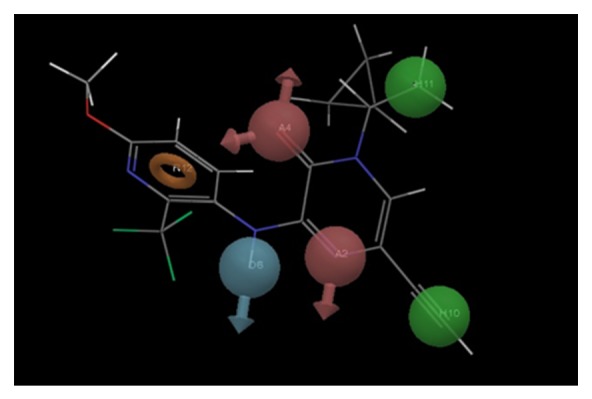
Best pharmacophore model AADHHR.47 aligned with molecule 48. Pharmacophore features are color coded: 2 hydrogen bond acceptors (A2, A4; pink), 1 hydrogen bond donor (D6; blue), 2 hydrophobic regions (H10, H11; green), and 1 aromatic ring (R12; orange).

Table 5 shows the fitness score for all the molecules of training set.  Figure 3 shows the AADHHR.47 aligned with ligand having maximum fitness score, that is, molecule 48 of the training set. Beside this survival score analysis, another validation method to characterize the quality of AADHHR.47 is represented by its capacity for correct activity prediction of training set molecules. 

**Figure 4:**
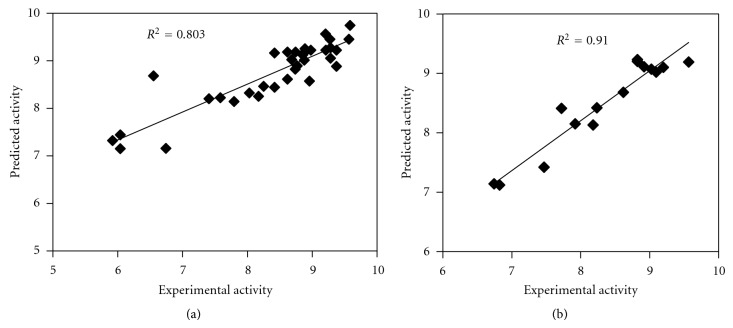
Relation between experimental and predicted CRF 1 antagonistic activity values of training set (a) and test set molecules (b) using model AADHHR.47.

The predicted CRF 1 antagonistic activity of training set molecule exhibited *R* ^2^ value of 0.803 (RMSD = 0.618) with experimental CRF 1 antagonistic activity using model AADHHR.47 (Figure 4). The validity and predictive character of AADHHR.47 was further assessed by using the test set prediction. 

The test set having fifteen molecules was analyzed. All the test set molecules were built and minimized as well as used in conformational analysis like all training set molecules. Then the activities of test set molecules were predicted using AADHHR.47 and compared with the actual activity. Actual and predicted activity values of test set molecules are given in Table 6. 

The predicted CRF 1 antagonistic activity of test molecule exhibited *R* ^2^ value of 0.91 (RMSD = 0.2961) with experimental CRF 1 antagonistic activity using model AADHHR.47 (Figure 4). For a reliable model, the squared predictive correlation coefficient should exceed 0.60 [17, 18]. The results of this study reveal that model AADHHR.47 can be used for the prediction of CRF 1 antagonistic activity. 

### 3.1. 3D QSAR Analysis

Additional insight into the CRF 1 antagonistic activity can be gained by visualizing the 3D QSAR model in the context of one or more ligands in the series with varying activity. This information can then be used to design new or more active analogues. 3D QSAR model based on the molecules of training and test set using various features, that is, hydrogen bond acceptor (A), hydrogen bond donor (D), hydrophobic regions (H), and aromatic ring (R) has been studied. 

#### 3.1.1. Hydrogen Bond Donor Field Predictions

**Figure 5:**
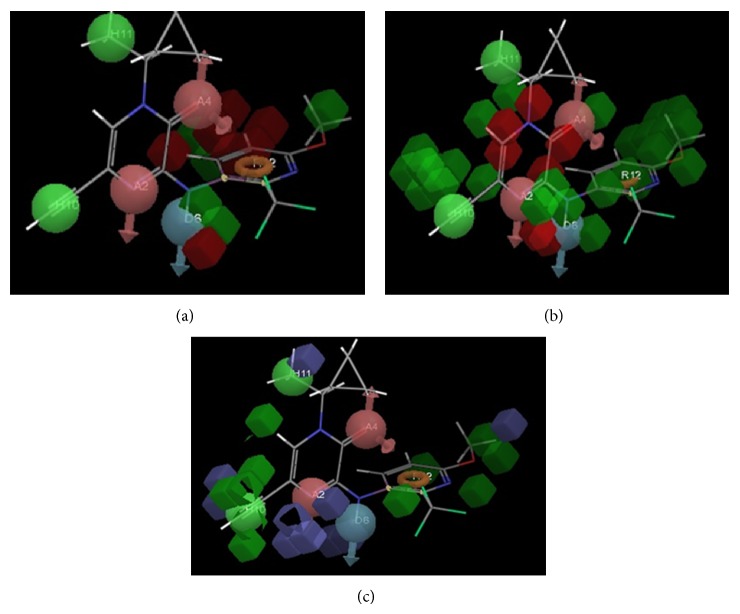
3-D QSAR model based on molecule 48 of training set illustrating hydrogen bond donor feature (a), hydrogen bond acceptor feature (b) and hydrophobicity feature (c).

The 3D QSAR model based on molecule 48 of the training set using hydrogen bond donor feature is shown in Figure 5(a). Red region near and around the meta- and parahydrogen of pyridine ring substituted at position 3 through NH terminal indicates that the substitutions at these positions by groups having more hydrogen bond donor property favor the CRF 1 antagonistic activity. Green region around the double bond between C_5_ and C_6_ on pyridine ring, NH terminal connecting pyrazinone and pyridine ring, methoxy group at C_4_ on pyridine ring, indicates that substitutions at these positions by groups having hydrogen bond donor property do not favor CRF 1 antagonistic activity.

#### 3.1.2. Hydrogen Bond Acceptor Field Predictions

The 3D QSAR model based on molecule 48 of the training set using hydrogen bond acceptor feature is shown in Figure 5(b). Red region around N1, carbonyl group at C_2_ and in between C_2_ and C_3_, double bond between C_5  _and C_6  _(all 3 on pyrazinone ring), indicates that the substitutions at these positions by groups having more hydrogen bond acceptor property favour the CRF 1 antagonistic activity. Green region around C_5_ on pyrazinone ring, NH terminal and methoxy group on pyridine ring, indicates that the substitutions at these positions by groups having more hydrogen bond acceptor property do not favour the CRF 1 antagonistic activity.

#### 3.1.3. Hydrophobicity Field Prediction

The 3D QSAR model based on molecule 48 of the training set using hydrophobicity feature is shown in Figure 5(c). Green region around ethynyl group substituted at position 5, at C_6_ (both on pyrazinone ring), double bond between C_5_ and C_6_, methoxy group at C_4_ (both on pyridine ring), indicates that the substitutions at these positions by groups having more hydrophobicity favour CRF 1 antagonistic activity and substitutions at these positions by more hydrophobic groups will result in increase in CRF 1 antagonistic activity. Blue region around methyl substituent of N^1^, position 4 (both on pyrazinone ring), methoxy group at C_4_ on pyridine ring, indicates that groups having more hydrophobic property do not favour CRF 1 antagonistic activity. 

## 4. Conclusions

This study shows the generation of a pharmacophore model AADHHR.47 for N^3^  phenylprazinones acting as CRF 1 antagonists. Pharmacophore modelling correlates activities with the spatial arrangement of various chemical features. The first hypothesis AADHHR.47 is the best hypothesis in this study, characterized by the best regression coefficient (0.803), degree of freedom (138.5), and highest survival score (2.980). Hypothesis AADHHR.47 represents the best pharmacophore model for determining CRF 1 antagonistic activity. AADHHR.47 consists of two hydrogen bond acceptors, one hydrogen bond donor, two hydrophobic regions, and one aromatic ring features. AADHHR.47 model had strong correlation between experimental and estimated activity of the training (*R* ^2^ = 0.803) and test (*R* ^2^ = 0.91) set molecules. Thus, AADHHR.47 pharmacophore model was able to accurately predict CRF 1 antagonistic activity, and the validation results also provide additional confidence in the proposed pharmacophore model. The obtained results suggested that the proposed 3D-QSAR model AADHHR.47 can be useful to rationally design new N^3^-phenylprazinones molecules as CRF 1 antagonists and also to identify new promising molecules as CRF 1 antagonists in large 3D database of molecules.

## Figures and Tables

**Table 1:** Various substituents attached to basic structure of N^3^-phenylpyrazinones.

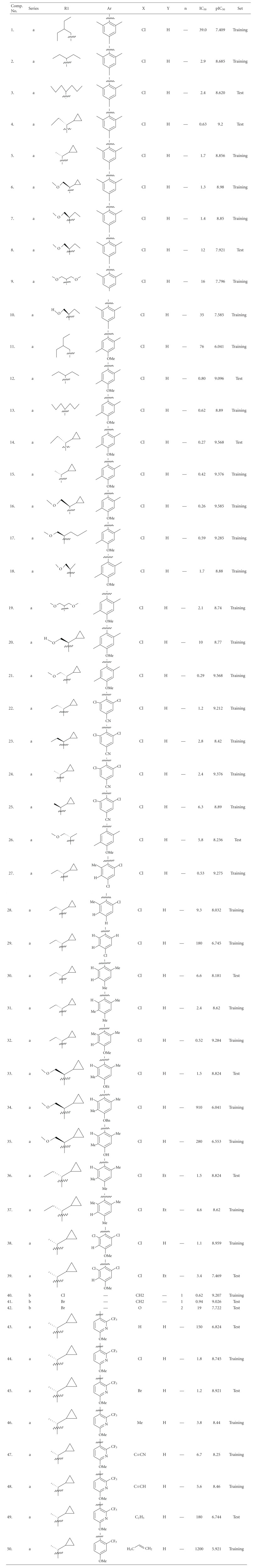

**Table 2:** Parameters of six featured pharmacophore hypothesis.

Serial number	Hypothesis	Survival score	*R* ^2^	*F*
1	AADHHR.47	2.980	0.803	138.5
2	ADHHHR.203	2.892	0.789	129.7
3	DHHHRR.611	2.863	0.7565	105.6
4	AAHHHR.19	2.700	0.7747	118.1

**Table 3:** Distances between different sites of model AADHHR.47.

Site-1	Site-2	Distances (A°)	Site-1	Site-2	Distances (A°)
A2	A4	3.572	A4	R12	3.474
A2	D6	2.520	D6	H10	5.541
A2	H10	3.291	D6	H11	6.688
A2	H11	4.947	D6	R12	3.243
A2	R12	5.054	H10	H11	5.984
A4	D6	3.850	H10	R12	8.343
A4	H10	6.431	H11	R12	7.161
A4	H11	3.802			

**Table 4:** Angles between different sites of model AADHHR.47.

Site-1	Site-2	Site-3	Angle (°)	Site-1	Site-2	Site-3	Angle (°)
A4	A2	D6	76.2	A2	H10	A4	21.3
A4	A2	H10	139.1	A2	H10	D6	15.3
A4	A2	H11	49.9	A2	H10	H11	55.8
A4	A2	R12	43.4	A2	H10	R12	1.5
D6	A2	H10	144.6	A4	H10	D6	36.6
D6	A2	H11	123.9	A4	H10	H11	35.4
D6	A2	R12	32.9	A4	H10	R12	22.8
H10	A2	H11	90.9	D6	H10	H11	70.8
H10	A2	R12	177.5	D6	H10	R12	13.8
H11	A2	R12	91.4	H11	H10	R12	57.2
A2	A2	D6	39.5	A2	H11	A4	45.9
A2	A4	H10	19.6	A2	H11	D6	18.2
A2	A4	H11	84.2	A2	H11	H10	33.4
A2	A4	R12	91.7	A2	H11	R12	44.9
D6	A4	H10	59.0	A4	H11	D6	29.3
D6	A4	H11	121.9	A4	H11	H10	78.7
D6	A4	R12	52.3	A4	H11	R12	9.7
H10	A4	H11	65.9	D6	H11	H10	51.5
H10	A4	R12	111.2	D6	H11	R12	26.8
H11	A4	R12	159.6	H10	H11	R12	78.2
A2	D6	A4	64.3	A2	R12	A4	44.9
A2	D6	H10	20.1	A2	R12	D6	25.0
A2	D6	H11	37.9	A2	R12	H10	1.0
A2	D6	R12	122.1	A2	R12	H11	43.7
A4	D6	H10	84.4	A4	R12	D6	69.8
A4	D6	H11	28.9	A4	R12	H10	45.9
A4	D6	R12	57.9	A4	R12	H11	10.7
H10	D6	H11	57.7	D6	R12	H10	24.0
H10	D6	R12	142.2	D6	R12	H11	68.4
H11	D6	R12	84.7	H10	R12	H11	44.6

**Table 5:** Experimental and predicted IC_50_ values of training set molecules based on hypothesis AADHHR.47.

Comp. No.	Experimental activity (IC_50_)	Predicted activity (IC_50_)	Fitness score	Comp. No.	Experimental activity (IC_50_)	Predicted activity (IC_50_)	Fitness score
1.	7.409	8.2	1.96	24	9.376	8.88	2.37
2.	8.685	9.02	2.14	25	8.89	9.25	1.29
5.	8.856	9.12	2.26	27	9.275	9.45	1.31
6.	8.98	9.22	1.67	28	8.032	8.32	1.46
7.	8.85	9.13	1.90	29	6.745	7.156	1.33
9.	7.796	8.14	2.01	31	8.62	8.61	1.37
10.	7.585	8.22	1.56	32	9.284	9.05	1.43
11.	6.041	7.15	1.10	34	6.041	7.44	1.31
13.	8.89	9.18	1.89	35	6.553	8.68	1.55
15.	9.376	9.22	2.48	37	8.62	9.18	2.08
16.	9.585	9.74	1.66	38	8.959	8.57	2.00
17.	9.285	9.27	1.75	40	9.207	9.56	1.92
18.	8.88	9.01	2.03	44	8.745	9.18	2.68
19.	8.74	8.82	2.01	46	8.421	8.44	2.60
20.	8.77	8.89	1.79	47	8.174	8.25	2.59
21.	9.568	9.45	1.43	48	8.252	8.46	3.00
22.	9.212	9.22	2.35	50	5.921	7.32	1.08
23.	8.42	9.16	1.25				

**Table 6:** Experimental and predicted IC_50_ values of test set molecules based on hypothesis AADHHR.47.

Comp. No.	Experimental activity (IC_50_)	Predicted activity (IC_50_)	Fitness score	Comp. no.	Experimental activity (IC_50_)	Predicted activity (IC_50_)	Fitness score
3.	8.620	8.68	1.99	36	8.824	9.23	1.95
4.	9.200	9.10	2.23	39	7.469	7.42	1.89
8.	7.921	*8.15*	2.01	41	9.026	9.07	1.81
12.	9.096	9.02	2.27	42	7.722	8.41	1.94
14.	9.568	9.19	2.23	43	6.824	7.12	2.51
26.	8.236	8.42	2.21	45	8.921	9.11	0.87
30.	8.181	8.13	1.18	49	6.744	7.14	2.61
33.	8.824	9.20	1.75				
